# The early educational environment at five years of age in a European cohort of children born very preterm: challenges and opportunities for research

**DOI:** 10.1186/s12887-024-04792-1

**Published:** 2024-05-29

**Authors:** Alyssa Smith-Longee, Samantha Johnson, Adrien M. Aubert, Anna-Veera Seppänen, Veronique Pierrat, Michael Zemlin, Jo Lebeer, Iemke Sarrechia, Veronica Siljehav, Jennifer Zeitlin, Mariane Sentenac, On behalf of the SHIPS research group, P. Van Reempts, E. Bruneel, E. Cloet, A. Oostra, E. Ortibus, K. Boerch, L. Huusom, P. Pedersen, T. Weber, L. Toome, H. Varendi, M. Männamaa, P. Y. Ancel, A. Burguet, P. H. Jarreau, P. Truffert, R. F. Maier, B. Misselwitz, S. Schmidt, L. Wohlers, M. Cuttini, D. Di Lallo, G. Ancora, D. Baronciani, V. Carnielli, I. Croci, G. Faldella, F. Ferrari, F. Franco, G. Gargano, A. van Heijst, C. Koopman-Esseboom, J. Gadzinowski, J. Mazela, A. Montgomery, T. Pikuła, H. Barros, R. Costa, LMendes Graça, M. do Céu Machado, C. Rodrigues, T. Rodrigues, U. Aden, A. K. Edstedt Bonamy, M. Norman, E. S. Draper, E. M. Boyle, A. Fenton, S. J. Johnson, B. N. Manktelow, D. W. A. Milligan, S. Mader, N. Thiele, J. M. Walz, S. Petrou, M. Bonet, C. Bonnet, REl Rafei, A. Piedvache, A. V. Seppanen

**Affiliations:** 1 Université Paris Cité, Inserm, INRAE, Centre for Research in Epidemiology and Statistics (CRESS), Obstetrical Perinatal and Pediatric Epidemiology Research Team, EPOPé, Paris, France; 2 Department of Health Sciences, University of Leicester, Leicester, UK; 3 Department of Neonatalogy, CHI Créteil, Créteil, F-94028 France; 4 Department of General Paediatrics and Neonatology, Saarland University, Saarland University Medical School, Homburg, Germany; 5 Department of Medicine & Population Health, Faculty of Medicine & Health Sciences, University of Antwerp, Antwerp, Belgium; 6 Department of Women’s and Children’s Health, Karolinska Institutet, Stockholm, Sweden; 7 Astrid Lindgren Children’s Hospital, Karolinska University Hospital, Stockholm, Sweden

**Keywords:** Very preterm birth, Early education, Europe, Inclusive education, Follow-up

## Abstract

**Background:**

Early childhood education offers opportunities for stimulation in multiple developmental domains and its positive impact on long-term outcomes and wellbeing for children is well documented. Few studies have explored early education in children born very preterm (VPT; <32 weeks of gestation) who are at higher risk of neurodevelopmental disorders and poor educational outcomes than their term-born peers. The purpose of the study is to describe and compare the educational environment of children born VPT in European countries at 5 years of age according to the degree of perinatal risk.

**Methods:**

Data originated from the population-based Screening to Improve Health In very Preterm infants (SHIPS) cohort of children born VPT in 2011/2012 in 19 regions from 11 European countries. Perinatal data were collected from medical records and the 5-year follow-up was conducted using parental questionnaires. Outcomes at 5 years were participation in early education (any, type, intensity of participation) and receipt of special educational support, which were harmonized across countries.

**Results:**

Out of 6,759 eligible children, 3,687 (54.6%) were followed up at 5 years (mean gestational age 29.3 weeks). At 5 years, almost all children (98.6%) were in an educational program, but type (preschool/primary), attendance (full-time/part-time) and use and type of school support/services differed by country. In some countries, children with high perinatal risk were more likely to be in full-time education than those with low risk (e.g. Estonia: 97.9% vs. 87.1%), while the inverse pattern was observed elsewhere (e.g. Poland: 78.5% vs. 92.8%). Overall, 22.8% of children received special educational support (country range: 12.4–34.4%) with more support received by children with higher perinatal risk. Large variations between countries remained after adjustment for socio-demographic characteristics.

**Conclusions:**

There are marked variations in approaches to early education for children born VPT in Europe, raising opportunities to explore its impact on their neurodevelopment and well-being.

**Supplementary Information:**

The online version contains supplementary material available at 10.1186/s12887-024-04792-1.

## Background

The number of children surviving very preterm birth (VPT; <32 weeks of gestation) continues to increase each year with advances in obstetric and neonatal medicine [1]. These children face higher risk of developmental difficulties than their term-born peers, including deficits in cognitive, behavioral, motor, and emotional functioning, language delays and sensory impairment [2–11]. In past decades, research on the developmental consequences of VPT birth primarily focused on severe impairments, such as cerebral palsy, blindness and deafness and severe cognitive deficits. However, research has broadened in scope to encompass minor and moderate motor, cognitive and behavioral difficulties [10, 12], and their effect on health, learning and quality of life [13]. Some research suggests that educational outcomes, motor outcomes and executive function may have worsened over time, calling attention to the need to better characterize, prevent and manage the long-term consequences of VPT birth [14, 15]. 

The health and development of the child plays an important role in determining the child’s readiness to enter the school system [16–18], and can impact their school performance [19] and social participation [20]. Studies have documented that children born VPT are at an increased risk of poorer academic attainment [19–22], that may persist throughout their schooling [23]. Due to the nature and severity of their neurodevelopmental delays, these children are more likely to have special educational needs [21, 22, 24], requiring educational support and assistance at school age [20, 21, 24–26], such as learning support, and speech and motor therapies [25, 27]. While medical research in the field of preterm birth remains paramount, there are calls for more attention to environmental factors that might promote these children’s longer term development and notably in education and schooling [28]. 

The period of early childhood is a critical window for both brain growth and development, during which children are highly sensitive to their environment and the people surrounding them [29, 30]. There is evidence that the family context and sociodemographic factors have an influence on the child’s development at an early stage [31, 32]. The nature of stimulation, especially within the family, may affect the child’s early development. More than any other factor, maternal education was found to predict motor, language and cognitive functions among children born preterm [33–35]. In addition to the family context, early childhood educational activities and programs offer opportunities for children to increase their skills and to be stimulated in multiple developmental domains before primary school age. They have been shown to play an important role in positively impacting children’s academic preparedness, cognitive, and social development in studies of children in the general population [31, 32, 36]. Participation in such activities and programs also provides the opportunity for early assessment and identification of needs allowing provision of early educational support and services to improve developmental outcomes [37], participation and success in primary school. Although “education” is one area identified as high priority for future research by clinicians, parents and individuals born preterm [28], very little empirical data exist on the early educational environment of children born VPT across diverse European contexts.

The principle of inclusive education, which aims to meet the educational needs of all children within a regular environment, regardless of their social or health circumstances, is recognized as instrumental for full economic and social participation in society [38]. Achieving inclusive and quality education is one of the United Nation’s sustainable development goals. However, policies and systems related to early childhood education and inclusion are very diverse across countries, and the reality of educational practices remains poorly documented. For instance, while primary education is compulsory from age 6 in most of European countries, the starting age of compulsory education is 4–5 years in United Kingdom and The Netherlands, and 7 in Estonia [36]. The settings where early childhood educational activities/programs take place (i.e. childcare- or education-type setting), the age from which a place is guarantee (from 6 months in Denmark to 5 years in the Netherlands), and measures to promote inclusiveness are additional components of national educational systems that might lead to variability in participation in educational activities before compulsory education [36]. 

International comparisons have the potential to further our understanding about how early educational practices influence long-term outcomes, well-being, learning and behaviors skills in children born VPT; however, to our knowledge, there is no prior literature comparing early childhood educational activities and programs across countries in the VPT population and there is a lack of a harmonized framework or common indicators to explore this question. Therefore, we aimed to define meaningful indicators for comparing the educational environment of 5-year-old children born VPT in 11 European countries.

## Methods

### Study design and population

We analyzed data from the Screening to Improve Health In Very Preterm Infants (SHIPS) project [39], which is the 5-year follow-up of the prospective population-based Effective Perinatal Intensive Care in Europe (EPICE) cohort of children born from 22 + 0 weeks to 31 + 6 weeks of gestation in 2011/2012 in 19 regions in 11 European countries: Belgium (Flanders); Denmark (the Eastern Region); Estonia (entire country); France (Burgundy, Ile-de-France and the Northern regions), Germany (Hesse and Saarland); Italy (Emilia-Romagna, Lazio and Marche regions); the Netherlands (Central and Eastern regions); Poland (Wielkopolska); Portugal (Lisbon and Northern regions); Sweden (Stockholm County) and the United Kingdom (UK) (East Midlands, Northern and Yorkshire and the Humber regions). Perinatal information was obtained from medical records in obstetric and neonatal units. At 5 years of age, data were collected using a parent-report questionnaire. Among the 6,759 children eligible to follow-up at 5 years, 3,687 (54.6%) participated in the study. A complete flowchart outlining the follow-up sample at 5 years is available in Additional file 1.

Ethical approvals were obtained in each country as required by national legislation. The European study was also approved by the French Advisory Committee on Use of Health Data in Medical Research and the French National Commission for Data Protection and Liberties. Informed consent to participate was provided by parents of children in the follow-up study.

### Measures

#### Measurement of early childhood education

At the 5-year follow-up, using standardized pretested questions in each country, parents were asked if their child was currently participating in any educational program (yes/no) and, if yes, the type of educational program in free-text responses, except for France and Italy, where prespecified options were provided (childcare, kindergarten/pre-school, primary school or special education). Parents were also asked to provide the age when their child started the educational program, whether their participation was full-time or part-time (intensity of participation) and whether their child received any educational support and services. Those responding yes were asked to provide details on the type of educational support or services with a free-text response, except in France where response options were proposed.

#### Sociodemographic and perinatal characteristics

We described the following sociodemographic characteristics: child sex, child’s age at follow-up, maternal age at delivery (< 25 years, 25–34 years, ≥ 35 years) and parity (primiparous/multiparous), socio-economic factors at the five-year follow-up, including maternal educational level (low, intermediate, or high) [33, 40], maternal cohabitating status (single versus married/cohabitating), and household employment status (employed/at least one parent unemployed).

Perinatal information from medical records included gestational age (GA) (23–24, 25–26, 27–28, 29–30 and 31 weeks) and birthweight (≥ 1000 g versus < 1000 g). Level of perinatal risk was determined using a composite variable, previously defined [33, 41], which classifies children with high perinatal risk (GA < 28 weeks and/or with a severe congenital anomaly and/or with a severe neonatal morbidity: bronchopulmonary dysplasia (oxygen or mechanical ventilation at 36 weeks postmenstrual age), retinopathy of prematurity stages III–V diagnosed before discharge, intraventricular hemorrhage grades III or IV, cystic periventricular leukomalacia or necrotizing enterocolitis needing surgery), low perinatal risk (GA 30–31 weeks, no congenital anomaly, no severe neonatal morbidity, and a normal birthweight for GA (*≥* 10th percentile for GA using intrauterine norms [42]), and medium risk (birth at 28–29 weeks GA without severe congenital anomaly or neonatal morbidity, or birth at 30–31 weeks with a birthweight < 10th percentile for GA and/or a non-severe congenital anomaly).

### Analysis

First, harmonized indicators across countries on the type of educational program and type of educational support and services (e.g. type and area of assistance) at five years were derived from parental reported free-text responses. In this first step, study partners from each country classified free-text responses into pre-defined categories (Additional file 2) and provided feedback regarding national specificities related to the proposed categories. To take into account differences in national educational systems, we further harmonized type of educational program based on the International Standard Classification of Education (ISCED, levels 0 and 1) [43]. in a final step, educational classifications were compared with the structure of the educational systems in each country according to the European Commission’s Eurydice report on Early Childhood Education and Care in Europe [36] to verify consistency and accuracy.

We then described the main characteristics of the study sample. The proportion of missing data for each indicator was examined. The distribution of the indicators of early childhood education (participation in any educational program, intensity of program, and reception of special educational support and services) was provided, stratified by country and by perinatal risk. We also described the distribution of the type of support services overall and by country. Then, we examined the variability in receiving special support and services by country, by perinatal risk and by level of maternal education taking into account sociodemographic characteristics in deriving predicted probabilities from generalized linear models with binomial distribution.

Analyses were performed on complete cases as less than 5% of missing data were reported for all study variables, except for intensity of participation (5.3%) and the free-text response on area of support and services (8.3% missing; 11.2% unable to be determined) (Additional file 3). All analyses accounted for the effects of potential bias due to selective attrition, using inverse probability weighting as done previously in this cohort [44]. Analyses were performed using Stata version 16 (StataCorp LLC, College Station, TX) and figures were generated using R version 4.1.2 and Microsoft Excel.

## Results

A description of the main sociodemographic and perinatal characteristics of the study sample (*n* = 3,687) is provided in Table 1. The median age of the children at the time of the survey was 5.5 years (ranging from 5.0 in Denmark to 5.7 in France, the Netherlands and the UK). Overall, 53.5% of children were male, and the majority had mothers with high or intermediate education (39.5% and 42.5%, respectively). The distribution of the level of perinatal risk was as follows: low (27.1%), medium (37.8%) and high (35.2%).

**Table 1:** Description of characteristics of children followed-up at 5 years

	**N**	**Raw** **%**	**Weighted**^b^ **%**
**Child characteristics**			
Child's age (years)	3,612	5.5 (4.3–7.2)^a^	
Male (%)	1,968	53.4	53.5
**Sociodemographic characteristics**			
Maternal age at delivery			
Less than 25 years	440	12.0	16.8
25–34 years	2,114	57.5	56.7
35 years or greater	1,123	30.5	26.5
Maternal educational level at 5 years			
Low	611	17.0	18.0
Intermediate	1,498	41.6	42.5
High	1,493	41.5	39.5
Single mother	436	12.2	13.5
At least one parent unemployed	399	11.2	11.7
**Perinatal characteristics**			
Gestational age			
23–24 weeks	132	3.6	3.6
25–26 weeks	483	13.1	11.6
27–28 weeks	847	23,0	22.2
29–30 weeks	1,274	34.6	35.2
31 weeks	951	25.8	27.4
Level of perinatal risk			
Low	922	25.7	27.1
Medium	1,362	37.9	37.8
High	1,311	36.5	35.2
Birthweight (grams)			
< 1000	1,099	29.8	27.7
Parity: Multiparous	1,448	39.7	43.0
**Country**			
Belgium (Flanders)	280	7.6	9.5
Denmark (Eastern Region)	152	4.1	4.3
Estonia (entire country)	134	3.6	2.1
France (Burgundy, Ile-de-France, Northern Region)	779	21.1	16.4
Germany (Hesse, Saarland)	280	7.6	10.0
Italy (Emilia-Romagna, Lazio, Marche)	693	18.8	14.3
The Netherlands (Central Eastern)	155	4.2	4.8
Poland (Wielkopolska)	189	5.1	3.7
Portugal (Lisbon, Northern Region)	433	11.7	8.9
The United Kingdom (East Midlands, Northern, Yorkshire and the Humber)	448	12.2	22.5
Sweden (Greater Stockholm)	144	3.9	3.4

^a^Median (Range)

^b^To account for the effects of potential bias due to selective attrition

### Qualitative report of the harmonization process of the indicators on early childhood education

#### Harmonization of type of educational program

The classification of free-text responses into pre-defined categories was limited in some countries due to use of generic terms by some parents that made it impossible to distinguish the level of education. For instance, in Portugal the term “colégio” is applied for private schools and could include pre-school, primary and secondary education. In these cases, the education type was marked as missing. The harmonization using the ISCED classification led to two main educational categories: (1) pre-primary educational activities and programs designing any programs which support children’s cognitive, physical, social and emotional development (ISCED 0; e.g. daycare institution, pre-school, kindergarten); and (2) primary education (ISCED 1) encompassing all activities providing children with fundamental skills in reading, writing and mathematics [43]. Thus, all children attending “børnehave” in Denmark and considered as a daycare center or “Kleuterschool” in Belgium were classified ISCED 0. And all children attending primary school were classified at ISCED level 1. A summarization of the final categories along with examples of free-text responses with English translation by country is provided in Additional file 4.

Information on special educational settings/school and personal education plans was also provided through free-text responses, however it was inconsistently reported across countries. For instance, we were unable to identify in the majority of countries if children had individual education plans, which aim to support their school curriculum in accordance to their educational needs. In some countries such as the UK, parents provided this information (example: “Educational health plan at school”; “EHC [*Education, health and care*] plan and one on one support”).

#### Harmonization of type and area of special support and services

Based on the parental free-text responses, we harmonized responses into four general areas of educational support/services (learning, speech, motor and emotional/behavioral) as shown in Table 2. The level of detail provided by parents made it possible to identify the domain in which support was provided, but we were not able to comprehensively classify the type of support/services (human vs. technical) or who was providing it (e.g., support teacher, personal aide, healthcare professional). For instance, generic answers given in the UK included “support in most lessons”; “Helped in a small group with teaching assistance”; “Intervention support”, as well as precise responses (i.e. “Speech and language”; “He has extra support in lessons (with reading, writing, numeracy)”; “Conductive education - class support includes physiotherapist, speech or language therapist, occupational therapist”). Regarding the area of support, it was also not possible to differentiate between sub-categories within our larger categories (e.g. fine vs gross motor assistance; sub-categories of learning assistance), as described in Additional file 5. For example, parents providing responses such as “occupational therapy”, “assistance with eating” or “gross and fine motor” were mapped to general motor assistance while those reporting “extra learning support within school” and “He has one-on-one mathematics sessions” were classified as learning support in general.

**Table 2:** Harmonization of free-text responses on area of special educational support/services^a^

Area of assistance provided^b^	n (%)	Type of assistance^c^	Examples of free-text responses from 5-year survey in national languages	English Translation
**Learning Assistance**	**270 (49.4%)**	Support Teacher Personal Assistant	Geïntegreerd Onderwijs (GON) Insegnante di Sostegno Assistente Educativo e Culturale (AEC) En resursperson Resurs i form av egen pedagog Apoio do docente de Educação Especial One to one support Teacher assistant Small group work	Integrated education Support teacher Personal school assistant Personal assistant Personal teacher Special education support teacher
**Speech and Language** **Services**	**218 (37.8%)**	Healthcare professional	Logopodie(a) Logopeed Terapia da fala Speech Therapist Speech & Language Therapy	Speech therapist Speech therapy
**Motor assistance and services**	**222 (38.0%)**	Personal assistant Technical assistance Healthcare professional	Fysioterapi Fysiotherapie Füsioteraapia Physiotherapy	Physiotherapy
			Ergotherapie Terapia ocupacional Occupational therapy	Occupational Therapy
			Rehabilitacja ruchowa Egen resurs: hjälp med att ta sig fram, mat, hygien, lek Grob- und feinmotorik Needs physical support e.g. when doing PE [*physical education*], carrying tray, using equipment	Physical rehabilitation Needs help from an assisting person in order to move, eat, personal hygiene etc Gross and fine motor skills
**Emotional, Social** **and Behavioral Support**	**114 (17.5%)**	Personal assistant Healthcare professional	Psycholog Psicologia Assistente Educativo e Culturale (AEC) IBT (intensiv beteendeträning) Assistenza alla comunicazione Social skills classes, additional support in class	Psychologist Psychologist Personal school assistant Intensive Behavioral Training Communication assistance

^a^Includes all countries except France; survey did not include this free-text question

^b^Classification of area and type of assistance is visually displayed in Fig. 2A

^c^ We were unable to consistently determine the type of assistance; however these were general trends identified during the harmonization process

### Description of the indicators on early childhood education at 5 years

**Fig. 1:**
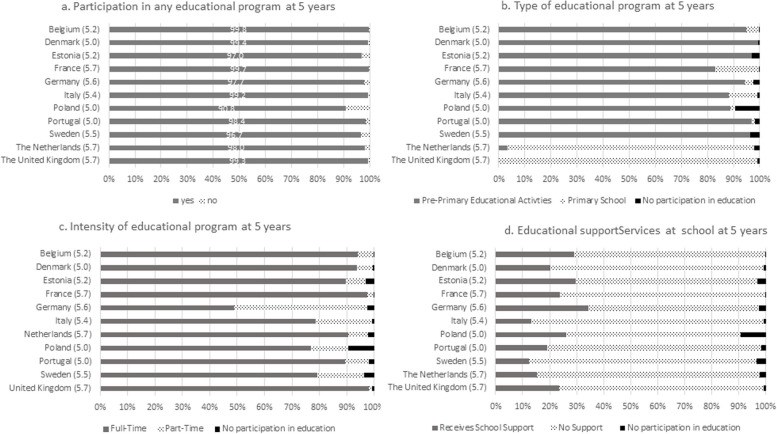
Distribution of the main early childhood education indicators by country

Overall, 98.6% of children were participating in an educational program at 5 years, variating from 90.8% in Poland to 99.8% in Belgium and France (Fig. 1a). The majority of children were attending pre-primary educational activities/programs in most countries (from 82.9% in France to 99.4% in Denmark), except in the UK and the Netherlands where the majority of children were enrolled in primary school (respectively, 99.2% and 94.4%) (Fig. 1b). Moreover, the majority of children were enrolled full-time in an educational program in all countries (from 76.7% in Poland to 98.1% in the UK) except in Germany (48.9%) (Fig. 1c). Overall, 22.8% of children participating in any educational program were receiving special support, however this proportion varied from 12.4% in Sweden to 34.4% in Germany (Fig. 1d).

**Fig. 2:**
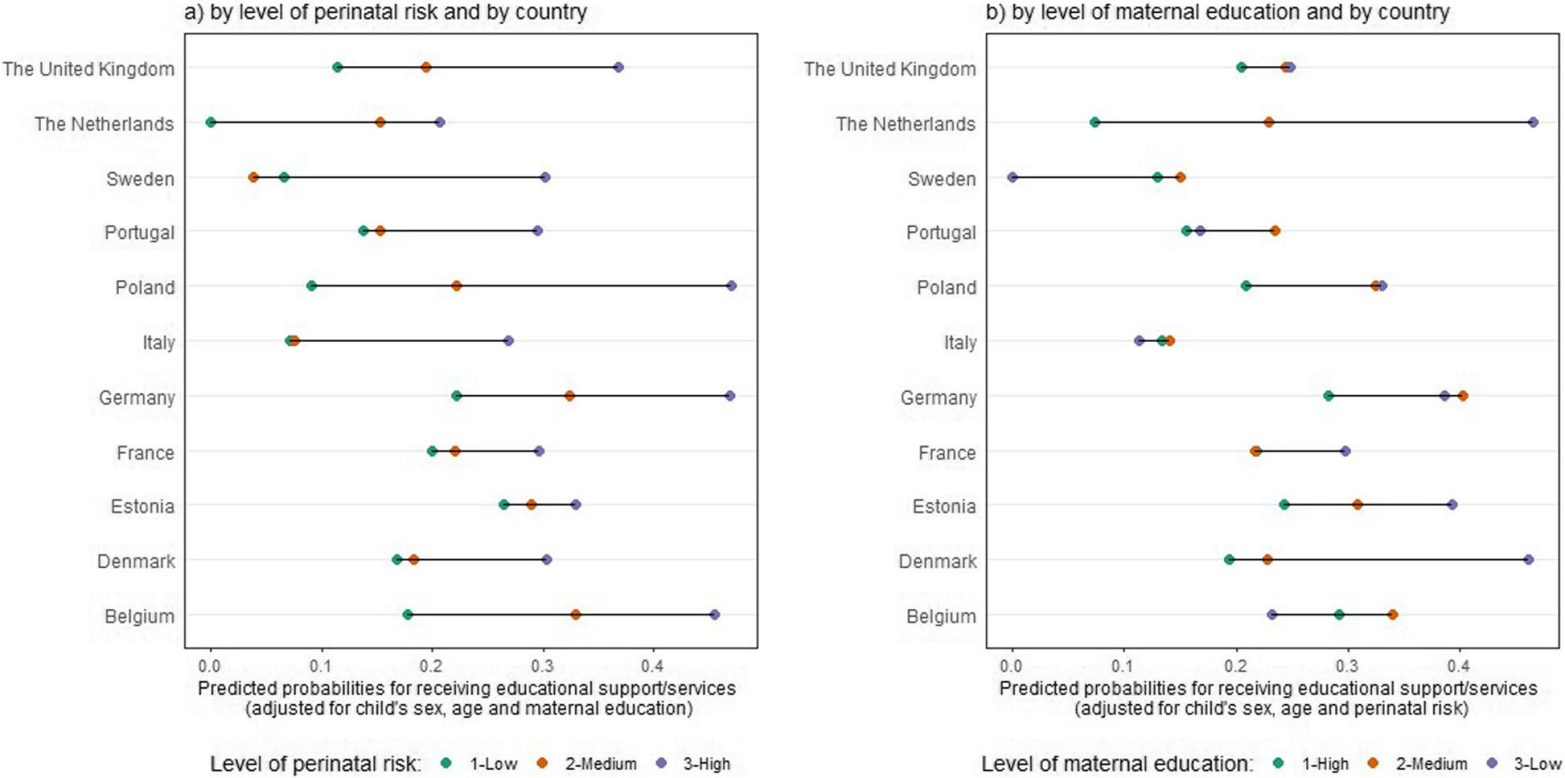
Reception of special support services by country and by level of perinatal risk (**a**) and by level of maternal education (**b**)

The indicators differed according to the level of perinatal risk (Table 3). Whether the participation in any educational program tended to decrease as level of perinatal risk increased in Poland and Sweden, no clear pattern was observed in other countries. Contrasting patterns with perinatal risk were observed in full-time attendance by country. In some countries, children with high perinatal risk were more likely to participate full-time than those with low perinatal risk (e.g. Estonia: 97.9% vs. 87.1%, respectively), while the inverse situation was observed in other countries (e.g. Poland: 78.5% vs. 92.8%, respectively). The proportion of children receiving special support increased as the level of perinatal risk increased in all countries. This proportion varied between 0% (the Netherlands) and 26.9% (Estonia) among those with low perinatal risk, and between 23.3% (the Netherlands) and 48.4% (Germany) among those with high perinatal risk. The variability in receiving special educational support according to the level of perinatal risk remained even after adjustment for child’s sex, age at the time of the survey, and maternal educational level (Fig. 2a). In addition, receiving special educational support was also found to vary by level of maternal education, with different patterns across countries (Fig. 2b). In most countries, however, children whose mothers had a lower educational level received more services, after adjusting for perinatal risk.

**Table 3:** Distribution of main early childhood education indicators at 5 years according to level of perinatal risk and country

	**Educational program participation** (n / % participating by level of perinatal risk)			**Intensity of participation in ECE**^**a**^ (n / % full time participation by level of perinatal risk)			**Special support**^**a**^ (n / % receiving support by level of perinatal risk)		
	**Among low** ***n* = 911**	**Among medium** ***n* = 1,331**	**Among high** ***n* = 1,231**	**Among low** ***n* = 873**	**Among medium** ***n* = 1,265**	**Among high** ***n* = 1,150**	**Among low** ***n* = 895**	**Among medium** **n = 1,316**	**Among high** **n = 1,218**
Belgium (Flanders)	93 (100)	84 (100)	77 (99.2)	89 (100)	73 (96.3)	58 (92.9)	15 (17.9)	29 (32.3)	31 (46.3)
Denmark (Eastern Region)	32 (97.5)	50 (100)	58 (100)	30 (94.7)	45 (96.0)	51 (92.5)	5 (14.5)	10 (18.8)	16 (29.4)
Estonia (entire country)	33 (100)	48 (94.1)	47 (97.9)	27 (87.1)	41 (97.7)	46 (97.9)	8 (26.9)	14 (30.6)	15 (32.8)
France (Burgundy, Ile-de-France, Northern Region)	173 (100)	321 (99.4)	240 (100)	166 (98.6)	303 (99.3)	219 (94.3)	33 (19.9)	67 (22.3)	68 (29.4)
Germany (Hesse, Saarland)	72 (98.8)	103 (96.4)	97 (98.4)	32 (46.1)	51 (56.5)	43 (46.0)	15 (22.1)	29 (32.7)	44 (48.4)
Italy (Emilia-Romagna, Lazio, Marche)	191 (99.5)	273 (98.7)	217 (99.4)	159 (83.9)	225 (83.2)	150 (69.4)	13 (6.9)	21 (7.6)	56 (27.3)
The Netherlands (Central Eastern)	24 (100)	47 (97.1)	73 (98.0)	23 (96.5)	43 (96.3)	69 (98.0)	0 (0.0)	5 (13.8)	13 (23.3)
Poland (Wielkopolska)	48 (96.3)	55 (91.9)	68 (86.4)	43 (92.8)	46 (85.5)	50 (78.5)	4 (9.2)	12 (21.1)	33 (48.4)
Portugal (Lisbon, Northern Region)	105 (100)	178 (97.1)	141 (98.7)	88 (92.3)	155 (95.5)	111 (88.4)	15 (15.9)	26 (14.9)	40 (28.8)
The United Kingdom (East Midlands, Northern, Yorkshire and the Humber)	111 (100)	126 (98.5)	167 (99.2)	107 (100)	119 (97.1)	150 (98.5)	12 (11.7)	26 (20.3)	61 (38.1)
Sweden (Greater Stockholm)	29 (100)	55 (98.5)	46 (92.1)	23 (100)	39 (88.9)	23 (66.06)	2 (5.4)	2 (3.0)	16 (33.3)
**Total**	**99.6**	**98.1**	**98.3**	**90.4**	**90.0**	**84.7**	**14.0**	**19.6**	**35.0**

^a^Among children enrolled in any educational program

### Description of the area of special educational support and services received at 5 years

Using the harmonized classification of support and services, 49% of children who had support at school received learning assistance, 38% received speech and language services, 38% motor assistance and services and 18% received emotional, social and behavioral support. However, different patterns were found by country, with the predominant area of support being learning in 6 out of 10 countries (UK, Netherlands, Italy, Germany, Belgium), while in other countries such as in Estonia and Portugal, it was speech and language services (Additional file 6).

## Discussion

This study offers a previously unavailable overview of the early childhood educational environment among children born VPT in European regions. We showed that most children were participating in an educational program at 5 years of age (98.6% of the overall sample). However, varying patterns (pre-primary/primary; intensity; special educational support) existed across European countries, likely due to different national policies and practices. There was a variation in the proportion of VPT children receiving educational support and services at school across countries from 12.4% in Sweden to 34.4% in Germany along with different patterns regarding to the area of assistance received. In some countries, receiving support in school was more related to level of perinatal risk and maternal education level.

Our findings reflect the heterogeneity in the structure of educational programs in Europe. For instance, in Denmark, educational activities before compulsory or primary school typically occur in center-based institutions outside schools while in other countries such as Belgium, Estonia and Poland, these are kindergarten-based or school-based programs. Moreover, and as suggested by our findings, kindergarten attendance in Germany on a part-time basis was a common practice at the time of the survey. We also showed that, at 5 years, most children in the Netherlands and the UK are already enrolled in primary education compared to other countries. While these children were older at the time of the survey (median: 5.7 years), this also reflects national policies as children typically transition to primary school at 4–5 years of age in these two countries [36]. If admission to primary school is mainly determined by the age of the child being 5–7 years old at the beginning of school year or the calendar year, there is some flexibility in some countries depending on the child’s development with the possibility of delaying the school entry at the request of parents of pre-primary setting (e.g. Italy, Portugal, Poland, Belgium). In Germany, readiness for school along with language ability, is a condition for admission to primary education and is systematically assessed [36]. And as for other rules, the age of compulsory schooling has been brought forward in several countries since the survey took place, such in France (3 years) and in Belgium (5 years).

We also showed a variation across countries in the proportion of children born VPT receiving any special support and services, and in the area of services/supports. It is well documented that children born VPT are at a higher risk of neurodevelopmental impairments or difficulties [7, 9, 10]. These children are also more likely to have special needs at school [19, 21], with prevalence of special educational needs increasing as GA decreases [26, 45]. Our study showed that receiving special support was related to level of perinatal risk, with slight differences according to country. This is expected given the higher rates of neurodevelopmental disabilities in this population related to lower GA and neonatal morbidities, such as bronchopulmonary dysplasia and intraventricular hemorrhage [46]. Possible explanations for the cross-country variation in receipt of special support/services relate to differences in national systems and policies, including (1) systematic evaluation of special education needs (e.g. Germany) which may allow learning problems to be identified and interventions put in place earlier than in other countries ; (2) inclusion policies which aim to provide all children with disabilities access to mainstream schools (e.g. Italy, Portugal, Sweden) in contrast to countries with more extensive networks of special educational settings [47]; (3) heterogeneous legislation and policies regulating and organizing the system for inclusive education and special support; for example, in Poland special educational needs are not defined in national legislation [47]. Differences in inclusive school contexts in Europe were suggested by a recent study that reported large variation in the school experiences of students reporting a chronic condition across countries [48]. 

The variation in support services at school may be explained by local resources or approaches to detecting problems [36]. In Estonia, the availability of specific support services is guaranteed for all children with services such as speech therapeutic and occupational therapy implemented at pre-school and school. In other countries (e.g. Italy, Poland), services are not mandatory at school, but are provided in most schools. Speech therapist is the most frequent specialists support provided at school, except in France where speech therapists are not present in educational settings [49]. Additionally, in some countries, children may receive these services outside of the school/educational system through the healthcare system instead. High healthcare use has been reported in this cohort in another study [40], where some of the healthcare services described by parents overlap with those reported in the questions around schooling. In some country, services such as motor development therapy, are mostly provided by healthcare systems than educational systems (e.g. Netherlands) [41]. Future comparative studies examining educational support services in European countries should also consider healthcare services provided outside school.

### Strengths and limitations

Strengths of this study are its large population-based prospective cohort design with geographic diversity on a topic that has not yet been studied across multiple countries, to our knowledge. Collaboration with research teams in 11 countries along with detailed reporting of the early childhood educational systems in Europe [36] and an available framework [43] for organizing and classifying information on education allowed us to describe the types of educational environments at 5 years of age (pre-primary education (ISCED 0) and primary education (ISCED 1)). We also identified four main areas of support services being provided at school (learning, speech, motor and emotional/behavioral). While we did not have access to an already pre-defined framework to harmonize this information, other studies from single countries on school-age VPT children report similar findings and supports services [22, 25, 27]. This work therefore provides foundational data for orienting future international research on this topic.

The study also had limitations. We relied on free-text questions to describe services received by the children because harmonized research questions do not exist for European countries. Thus, while we were able to use this data to describe the range of support services provided, parents responded with different levels of detail that limited our ability to derive some important indicators for the full sample, including special educational setting or whether children had individual education plans or to get more granularity around the type and area of support services. Additionally, the children included in our study were at the end of the early educational phase (5/6 years of age) and for the children receiving educational support services, we were unable to determine how long these children had been receiving this support. Also, results by country should be interpreted with caution due to small sample. Lastly, attrition bias could be present and may introduce selection biases leading to bias estimates if reasons for loss to follow-up are associated with early childhood educational environment and differ between countries.

However we used inverse probability weighting [44] based on baseline information available for all children to account for loss to follow-up in the cohort.

### Implications for future multinational questionnaires on schooling and education

This study provides results on the type of educational programs used by children born VPT and the area of support services provided in early educational programs derived from free-text responses from a parental questionnaire at 5 years. In this study, through the complex task of assessing and harmonizing free-text responses across 11 countries, we were able to identify four previously unavailable categories of domains of support that can be used in further international comparisons. For further investigation, more information could be collected on the support services or interventions the children received along with who provided them (e.g. teacher, one-to-one personal aide, a technical device, etc.), as on other additional special needs (e.g. special school settings, mainstream school with additional needs and/or special classes and/or personal education plan). These details could allow more granularity of the information provided by the parents. This information is even more important to collect given that most population-based cohorts of children born VPT carry out follow-up at five year-old [35] which is an important developmental milestone, but also a period with large structural heterogeneity regarding early education national systems.

## Conclusions

There is marked variation in early educational practices regarding children born VPT aged five years across European countries, especially regarding educational support and services provided at early childhood education. While developmental outcomes in children born VPT are well documented, our study emphasizes the importance of further investigating how developmental outcomes can be improved, such as through the educational environment, in order to meet the long-term developmental and educational needs of children born VPT.

### Supplementary Information

**Additional file 1. **Flow chart of the study sample selection.

**Additional file 2.** Classification of type of educational program and type of educational support and services: pre-defined categories.

**Additional file 3.** Percentages of missing data of main variables of participants followed up at 5 years and enrolled in an educational program (*n*=3565).

**Additional file 4.** Type of early childhood educational program at 5 years; Common free text responses in national language with English translation.

**Additional file 5.** Classification of free-text responses on area of special educational support/services received at 5 years.

**Additional file 6.** Distribution of area of special educational support/services at 5 years by country.

## Data Availability

The datasets generated and/or analysed during the current study are not publicly available due to stipulations in the original review board authorizations for the study, but anonymised data on the variables used in the study are available from the corresponding author on reasonable request.

## Abbreviations

VPT: Very preterm birth
GA: Gestational age
SHIPS: Screening to Improve Health In Very Preterm Infants
EPICE: Effective Perinatal Intensive Care in Europe
ISCED: International Standard Classification of Education
