# Supplementary material for: The early educational environment at five years of age in a European cohort of children born very preterm: challenges and opportunities for research
Source: BMC Pediatr. 2024 May 29;24:369. doi: 10.1186/s12887-024-04792-1 (PMC11134723; doi:10.1186/s12887-024-04792-1)
Supplement: Supplementary file 1 — Additional file 1. Flow chart of the study sample selection. [file 12887_2024_4792_MOESM1_ESM.docx]

**ADDITIONAL FILE**

| **Additional file 1.** Flow chart of the study sample selection | | | |  |
| --- | --- | --- | --- | --- |
|  |  |  |  |  |
|  | *Stillbirths, TOP and live births* | |  |  |
|  | 10329 | 100% |  |  |
|  |  |  |  |  |
|  |  |  | *Stillbirths, Termination of pregnancy* | |
|  |  |  | 2429 | 23.5% |
|  |  |  |  |  |
|  | *Live births* | |  |  |
|  | 7900 | 84.4% |  |  |
|  |  |  |  |  |
|  |  |  | *Unknown or missing* | |
|  |  |  | 2 | 0.03% |
|  |  |  |  |  |
|  |  |  | *Deceased before discharge* | |
|  |  |  | 1106 | 14.0% |
|  |  |  |  |  |
|  | *Alive at discharge* | |  |  |
|  | 6792 | 86.0% |  |  |
|  |  |  |  |  |
|  |  |  | *Deceased* | |
|  |  |  | 33 | 0.5% |
|  |  |  |  |  |
|  | *Alive at 5; invited to follow-up* | |  |  |
|  | 6759 | 99.4% |  |  |
|  |  |  |  |  |
|  |  |  | *Non-participants* | |
|  |  |  | 3072 | 45.5% |
|  |  |  |  |  |
|  |  |  |  |  |
|  | *Followed-up at 5 (final sample) ** | |  |  |
|  | 3687 | 54.6% |  |  |
|  |  |  |  |  |
