# Supplementary material for: The early educational environment at five years of age in a European cohort of children born very preterm: challenges and opportunities for research
Source: BMC Pediatr. 2024 May 29;24:369. doi: 10.1186/s12887-024-04792-1 (PMC11134723; doi:10.1186/s12887-024-04792-1)
Supplement: Supplementary file 2 — Additional file 2. Classification of type of educational program and type of educational support and services: pre-defined categories. [file 12887_2024_4792_MOESM2_ESM.docx]

**ADDITIONAL FILE**

**Additional file 2.** Classification

**Type of education**

1. Day care/crèche
2. Pre-school
3. Primary school
4. Special education settings
   1. if so, specify in english (special class or school)
5. Others
   1. if so, specify in english

**Type of special educational support and services**

1. Type of assistance
   1. Human
      1. Teacher or personal assistant
      2. Professional (health) are provider
      3. Other type of assistants
   2. Technical/ mechanical
2. Area of assistance provided
   1. Learning
      1. Language
      2. Reading
      3. Writing
      4. Numeracy
      5. Memory
      6. In general / not specified
   2. Motor
      1. Gross
      2. Fine
      3. Not specified
   3. Emotional / behavioral / Social
   4. Other (If so, please provide a translation in english)
3. Special school / personal education plan
   1. Special school
   2. Personal education plan
4. Comment
