# Supplementary material for: The early educational environment at five years of age in a European cohort of children born very preterm: challenges and opportunities for research
Source: BMC Pediatr. 2024 May 29;24:369. doi: 10.1186/s12887-024-04792-1 (PMC11134723; doi:10.1186/s12887-024-04792-1)
Supplement: Supplementary file 3 — Additional file 3. Percentages of missing data of main variables of participants followed up at 5 years and enrolled in an educational program (n=3565). [file 12887_2024_4792_MOESM3_ESM.docx]

**ADDITIONAL FILE**

**Additional file 3.** Percentages of missing data of main variables of participants followed up at 5 years and enrolled in an educational program (n=3565)

|  | | **n of missing** | **% of missing** | |  |  |  |  |  |  |  |
| --- | --- | --- | --- | --- | --- | --- | --- | --- | --- | --- | --- |
| **Educational environment characteristics** |  |  |  | |  |  |  |  |  |  |  |
| Type of education |  | 119 | | 3.3 | | | | |  | |  |
| Reception of special education provision |  | 46 | | 1.3 | | | | |  | |  |
| Intensity of program |  | 190 | | 5.3 | | | | |  | |  |
| *Area of support services |  | 115 | | 19.5 | | | | |  | |  |
| **Socioeconomic and demographic characteristics** |  |  | |  | | | | |  | |  |
| Maternal Education | 58 | | | 1.7 | | | | | |  |  |
| Maternal Cohabitating status | 46 | | | 1.3 | | | | | |  |  |
| Household employment status | 60 | | | 1.7 | | | | | |  |  |
| Child sex | 0 | | | 0.0 | | | | | |  |  |
| Child age at survey | 75 | | | 2.0 | | | | | |  |  |
| Maternal country of birth | 16 | | | 0.5 | | | | | |  |  |
| Maternal age at birth | 9 | | | 0.3 | | | | | |  |  |
| **Perinatal characteristics** |  |  | |  | | | | |  | |  |
| Gestational age | 0 | | | 0.0 | | | |  | | | |
| Perinatal risk | 90 | | | 2.6 | | | |  | | | |
| Birthweight | 0 | | | 0.0 | | | |  | | | |
| Parity | 40 | | | 1.1 | | | |  | | | |
| **Among those receiving support (n=591); excludes France; 34/115 responses were missing and 81/115 were responses that we were unable to determine the area of support services* | | | | | | | |  | | | |
|  |  | | |  | | | |  | | | |
