# Supplementary material for: The early educational environment at five years of age in a European cohort of children born very preterm: challenges and opportunities for research
Source: BMC Pediatr. 2024 May 29;24:369. doi: 10.1186/s12887-024-04792-1 (PMC11134723; doi:10.1186/s12887-024-04792-1)
Supplement: Supplementary file 4 — Additional file 4. Type of early childhood educational program at 5 years; Common free text responses in national language with English translation. [file 12887_2024_4792_MOESM4_ESM.docx]

**ADDITIONAL FILE**

**Additional file 4.**  Type of early childhood educational program at 5 years; Common free text responses in national language with English translation

|  |  | **Belgium** | **Denmark** | **Estonia** | **France** | **Germany** | **Italy** | **The Netherlands** | **Poland** | **Portugal** | **Sweden** | **The UK-England** |
| --- | --- | --- | --- | --- | --- | --- | --- | --- | --- | --- | --- | --- |
| **Pre-school educational activities (ISCED 0)** | Centre-based institutions including educational activities | n/a | **3-5 yo** | n/a | n/a | **3-6 yo** | n/a | n/a | n/a | n/a | **1-6 yo** | n/a |
|  |  |  | Børnehave (Kindergarten; daycare institution) |  |  | Kindergarten (not a part of the regular public school) |  |  |  |  | Förskola Dagis (Pre-school center) |  |
|  | School-based programme in school settings | **2 ½-6 yo** | n/a | **1 ½ -7 yo** | **2-6 yo** | **5-6 yo** | **3-6 yo** | **6-8 weeks - 4 years** | **3-7 yo** | **3-6 yo** | **6-7 yo** | **3-4 yo** |
|  |  | Kleurterschool   Kleuterklas  Kleuteronderwijs Maternelle Kleuteronderwijs (Pre-School) |  | Kindergarten (Pre-School) | Ecole Maternelle Maternelle: Petits, Moyens, Grands  (Kindergarten) | Vorschule Vorklasse (school-based programme in some *Länder*) | Kindergarten | (Voorschoolse educatie en kinderopvang) (Kindergarten) Peuterspeelzaal Pre-School) | Przedszkole (Kindergarten) Zerówka (Pre-School) | Pré-escola (Pre-School) Jardim de Infância Infantário (Kindergarten) | Förskoleklass (Pre-primary preschool class) | Reception and Nursery classes in schools (Pre-School) |
| **Primary compulsory school (ISCED 1)** | | **6-12 yo** | n/a | **7-16 yo** | **6-11 yo** | **6-10 yo** | **6-11 yo** | **4-12 yo** | **7-15 yo** | **6-12 yo** | **7-16 yo** | **5-11 yo** |
|  |  | Lagere School Basisschool (Primary School) |  | Põhikool (Primary School) | Cours Préparatoire de l’école Primaire CP (Primary School) | Grundschule (Primary School) Ganztagsschule (All-day School) Schule (School) | Primary School | Basis onderwijs (Primary School) | Szkoła (Primary School) | Escola Primária (Primary School) | Grundskola (Primary School) | Primary Pre-prep Infant (Primary schools) |

**Note**: n/a= not available; yo=years old; ISCED=International Standard Classification of Education 2011

**Additional file 5**. Classification of free-text responses on area of special educational support/services received at 5 years


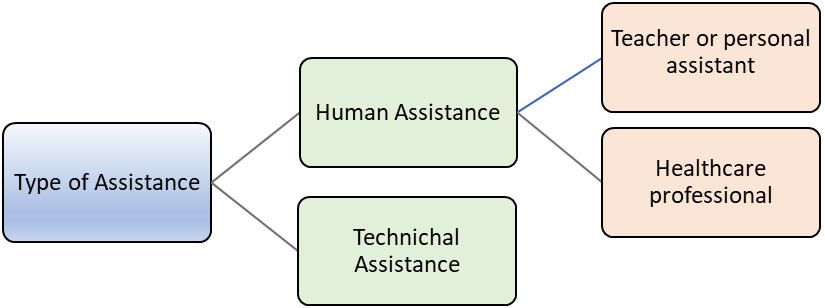

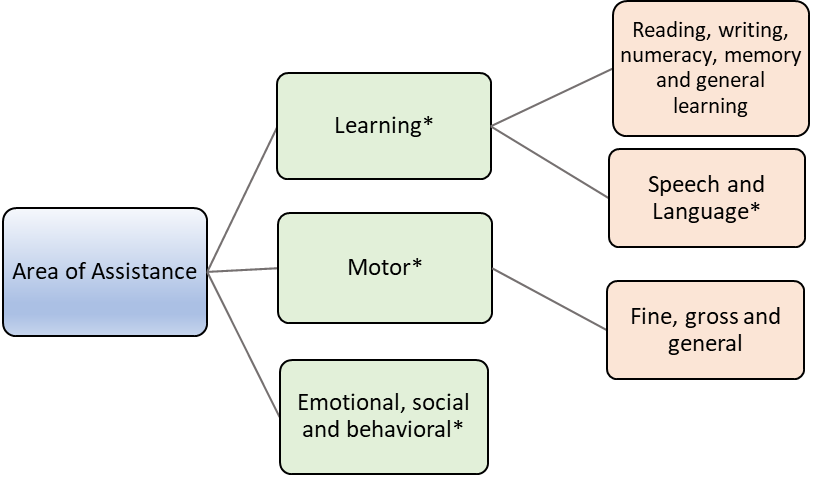


2nd Subcategories

1st Subcategories

Main Categories

**Note**: Categories and sub-categories used to classify the free-text responses on type of support services; only information on Area of assistance was used in this study

**Additional file 6**. Distribution of area of special educational support/services at 5 years by country

***Note:*** n=474; children may have more than one of the support or services; the free-text question on type of special educational support/services was not asked in France.
