# Supplementary material for: The early educational environment at five years of age in a European cohort of children born very preterm: challenges and opportunities for research
Source: BMC Pediatr. 2024 May 29;24:369. doi: 10.1186/s12887-024-04792-1 (PMC11134723; doi:10.1186/s12887-024-04792-1)
Supplement: Supplementary file 5 — Additional file 5. Classification of free-text responses on area of special educational support/services received at 5 years. [file 12887_2024_4792_MOESM5_ESM.docx]

**ADDITIONAL FILE**

**Additional file 5**. Classification of free-text responses on area of special educational support/services received at 5 years


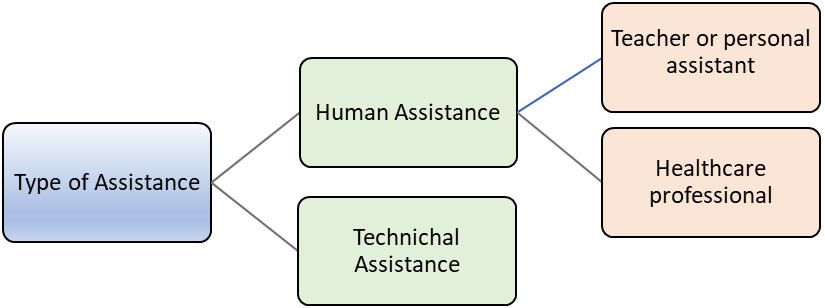

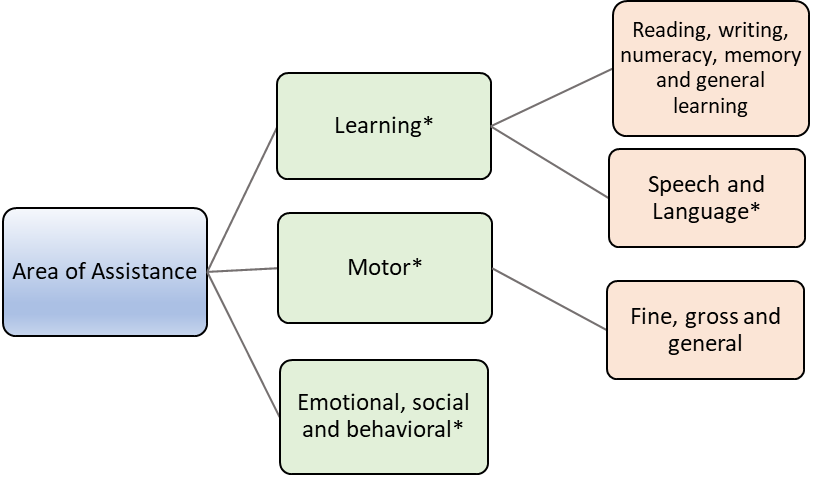


2nd Subcategories

1st Subcategories

Main Categories

**Note**: Categories and sub-categories used to classify the free-text responses on type of support services; only information on Area of assistance was used in this study
