# Supplementary material for: The early educational environment at five years of age in a European cohort of children born very preterm: challenges and opportunities for research
Source: BMC Pediatr. 2024 May 29;24:369. doi: 10.1186/s12887-024-04792-1 (PMC11134723; doi:10.1186/s12887-024-04792-1)
Supplement: Supplementary file 6 — Additional file 6. Distribution of area of special educational support/services at 5 years by country. [file 12887_2024_4792_MOESM6_ESM.docx]

**ADDITIONAL FILE**

**Additional file 6**. Distribution of area of special educational support/services at 5 years by country

***Note:*** n=474; children may have more than one of the support or services; the free-text question on type of special educational support/services was not asked in France.
